# Assessment of public knowledge and attitude toward antibiotics use and resistance: a community pharmacy-based survey

**DOI:** 10.1186/s40545-023-00619-z

**Published:** 2023-09-28

**Authors:** Abdulmuminu Isah, Azeez Babatunde Aina, Kenechukwu C. Ben-Umeh, Chinyere A. Onyekwum, Cynthia C. Egbuemike, Cheleolisa V. Ezechukwu, Daniel O. Umoru, Regina N. Nechi

**Affiliations:** 1 Department of Clinical Pharmacy and Pharmacy Management, University of Nigeria, Nsukka, Nigeria; 2 Department of Pharmacy Practice, Purdue University, 575, Stadium Mall Drive, West Lafayette, IN 47907 USA; 3 Department of Pharmacotherapy, College of Pharmacy, University of Utah, Salt Lake City, UT USA; 4 National Hospital, Abuja, Nigeria; 5 Biomedical Sciences, College of Medicine, Pennsylvania State University, Pennsylvania, USA

**Keywords:** Antibiotics, Attitude, Community pharmacy, Knowledge, Resistance

## Abstract

**Background:**

Antimicrobial resistance is a public health challenge affecting all aspects of healthcare systems. Policies to reduce antimicrobial resistance should be implemented and monitored in community pharmacies, because they are patients' first point of care. Public awareness of relevant knowledge and attitudes on antimicrobials is a feasible strategy to mitigate the spread of antimicrobial resistance by exploiting the relationship between pharmacists and patients in the community pharmacy setting. The study evaluated and determined predictors of antibiotic knowledge and attitudes toward antibiotic use and resistance in community pharmacy patients.

**Methods:**

A cross-sectional design was used to retrieve data in five randomly selected community pharmacies in Lagos and Abuja using a self-administered questionnaire. Descriptive and inferential statistics were utilized for characterizing and determining the associations between predictors and outcomes at *p* < 0.05. Logistic regression was used to identify predictors of patients’ knowledge and attitude to antibiotic use*.*

**Results:**

A total of 964 clients participated in the study: 526(54.7%) were females, and 358(37.3%) were aged 25–34. chlorpheniramine–maleate and levonorgestrel were wrongly identified as antibiotics by 621 (64%) and 490 (50%) respondents, respectively. Many respondents, 448(46.5%), strongly agree that antibiotic creams should be mixed with body creams. The result of the multivariable logistic regression showed secondary education [Odds Ratio (OR): 0.31, 95% CI 0.10–0.97, *p* value: 0.044], urban residence (OR: 1.45, 95% CI 1.01–2.08, *p* value: 0.043) and age 34 (OR: 1.55, 95% CI 1.01–2.37, *p* value: 0.045) were strong predictors of knowledge on antibiotics, while community pharmacy location (OR: 5.48, 95% CI 3.45–8.70, *p* value: ≤ 0.001), urban residence (OR: 2.57, 95% CI 1.67–3.96, *p* value: ≤ 0.001), and antibiotic recommender (OR: 0.55, 95% CI 0.35–0.85, *p* value: 0.008) were predictors of respondents’ attitude to antibiotic use.

**Conclusions:**

The study established that sociodemographic factors could impact community pharmacy clients' knowledge and attitude toward antibiotic use and resistance and should be considered when developing policies to curb the spread of resistant microbes. Community pharmacies should educate community pharmacy clients on the dangers associated with the misuse of antibiotics with stringent antibiotic stewardship programs and restrict access to antibiotics over-the-counter.

## Introduction

Advancement in science and technology has informed the growth of the human population over the century and the emergence of new diseases [1]. The increased interconnectivity due to efficient transportation networks among countries facilitate the rapid spread of infectious diseases and the appearance of resistant microorganisms [2]. The World Health Organization (WHO) defines infectious diseases as those transmittable diseases caused by viruses, parasites, bacteria, and fungi [3]. It was stated that antimicrobial resistance is one of the ten major health crises facing humanity with a negative impact on socio-economic status [4]. Outcomes from these diseases can reduce the quality of life, increase health costs, reduce life expectancy and ultimately lead to death. Infectious diseases are rife in low-and-middle-income countries and is reflected in their low life expectancy [5, 6]. Nigeria is one of only five members of the WHO African region to record five or more public health incidents annually, with over 20 infectious disease outbreaks and public health crises between 2016 and 2018 [7].

Antibiotics are prescription-only medicines in Nigeria according to the national guideline and should be dispensed under the supervision of a certified medical professional. Unfortunately, due to poor prescription monitoring, antibiotics are regularly dispensed over the counter in community pharmacies and patent proprietary medicines vendors (PPMVs) with little or no oversight [6]. The tendency for irrational use of medications is high in Nigeria and can lead to polypharmacy, antibiotic overuse, and non-adherence to medication [8].

Many factors have contributed to the prevalence of antimicrobial resistance including but not limited to the misuse and overuse of antimicrobials, lack of awareness, poor knowledge and lax public health policies [9]. The report of antibiotic misuse and abuse is not only found in hospital settings. Many cases have been reported in community settings worldwide, especially in developing countries [10]. Sociodeterminants of health, human behavior, and health policies play pivotal role in prescribing practices that contribute to antibiotic resistance [11]. Similarly, the pressure on the abuse and inappropriate use of antibiotics can be attributed to consumers' misconceptions about antibiotics which are evident in the public's cultural health practices.

Countries and regions worldwide need to develop a viable and reliable framework for monitoring and assessing the public's knowledge of antibiotic use and misuse to reduce resistance [12]. Such a framework must prioritize patients’ factors to achieve their upstream and downstream effects. Hence, this study assessed participants' knowledge and attitudes about the use of antibiotics while determining sociodemographic predictors that could be used to implement policies to reduce antibiotic resistance. It hypothesized that community pharmacy clients' characteristics would be predictors of antibiotic knowledge and attitude.

## Methods

### Design and study population

The study was a cross-sectional questionnaire-based survey conducted in five randomly selected community pharmacies in Lagos and Abuja, Nigeria. The data were collated over 3 months (July to September 2021) in five different districts: Utako (Abuja), Kubwa (Abuja), Yaba (Lagos), Victoria Island (Lagos), and Ajegunle (Lagos).

### Study location

The study was conducted in Lagos and Abuja. Lagos is the economic center of Nigeria and the most populous city in Sub-Saharan Africa, while Abuja is the Federal Capital Territory. Both locations have many community pharmacies dispersed in rural and urban areas. The percentage of urban to rural pharmacies used in this study was 60–40%. Three of the community pharmacies were in an urban setting, while 2 were in a rural location.

### Study size

The estimated study population for a month (average number of visitors to all the community pharmacies selected) was 10,000, from which a sample size of 370 was determined using the Raosoft Online Sample Size calculator, assuming a 5% error and 95% confidence interval. Data collection was conducted for 3 months to improve the robustness of the sample (1110). A proportionate distribution of 1110 was done among the four sites, although 964 respondents consented and completed the questionnaire (response rate of 86.8%).

### Eligibility criteria

Adult clients who visited the community pharmacies during the period of the study were randomly invited to participate in the study. Researchers verbally informed the clients about the ongoing study, and leaflets were presented to seek their interest. Interested participants were directed to read the consent information imprinted on the front page of the questionnaire carefully before filling it. Further interpretations were given to a few respondents who found it difficult to understand the information on the consent page. Only those who consented with their signature were given the study instrument to complete.

### Study instrument

A self-administered questionnaire with 2-point and 5-point Likert-scale domains was used to determine the knowledge and attitude of respondents on the use of antibiotics, respectively. The questionnaire was revised from previous studies and modified to assess the study’s research questions [13, 14].

Prior to data collection, the face validation of the instrument was done in collaboration with research experts from the University of Nigeria and community pharmacists practicing around the study settings. Researchers ensured that community pharmacists selected for face validation were not in proximity to the study sites and had no affiliation with them. Five willing participants were randomly selected to test the questionnaire at each test site. The content validation of the items of the adjusted questionnaire was determined using the viewpoint of the panel of clinical pharmacy experts according to the method outlined by Yusoff [15]. The instrument was sent to the 5 experts with a relevancy and clarity scale for rating. The ratings were reviewed for each item and section of the questionnaire resulting in a scale-level content validity index of 0.82 and 0.92 for the knowledge and attitude domains, respectively, based on the universal agreement method. The resulting scores were within the accepted range for content validity index [16].

The Likert scales to assess respondents’ attitudes were Strongly Disagree = 1, Disagree = 2, Neutral = 3, Agree = 4, and Strongly Agree = 5, with a minimum sum score of 13 and a maximum sum score of 65. Respondents who correctly answered questions on antibiotic knowledge were given a score of 1, while those who chose wrongly were scored 0. Correct identification of commonly used antibiotics was assessed with a Yes/No question using their generic and trade names. Any antibiotic that was wrongly identified was given a score of 0, while a score of 1 was for correct identification. Hence, the minimum sum of the score for knowledge was 0, and the maximum sum of score was 23.

### Data collection

The questionnaires were distributed to the respondents, self-administered, and retrieved from the respondents on the same day. The patients that visited the community pharmacies were approached, and those that gave their consent were administered the questionnaire. This was done on weekdays within the specified period of the survey. The filling exercise was adequately monitored to ensure prompt return. The questionnaire was made up of three sections:

1. Demographic and occupational characteristics of respondents.
2. Knowledge of antibiotic use and antibiotic resistance.
3. Attitude toward antibiotic use and resistance.

### Data analysis

The data collected was analyzed using Statistical Package for Social Sciences (version 28). Descriptive statistics was used to evaluate measures such as frequencies, percentiles, and means of the sociodemographic characteristics of respondents. Binomial logistic regression was also conducted as appropriate. Univariate analysis established the relationship between the sociodemographic predictors and outcomes. Multivariable logistic regression was used to determine the odds of outcomes between predictors and outcomes. All statistical values were set at a threshold *p* value of 0.05.

Negatively worded questions in the knowledge section of the questionnaire were adequately re-coded and transformed during analysis to ensure that they were correctly scored. Knowledge and attitude were dichotomized into poor and good, using the median of the respondents’ sum of scores. The sum of knowledge scores greater than or equal to 14 was categorized as good knowledge, while the sum of knowledge scores less than 14 was labeled poor knowledge. For attitude, a sum of score greater than or equal to 42 was categorized as good attitude, while a score less than 42 was poor attitude.

## Results

The total number of respondents that filled the questionnaires was 964. Descriptive analysis of the socio-demographic data (Table 1) showed that 358 (37%) of the respondents were between the ages of 25 and 34. Only 259 (26.9%) of the respondents used antibiotics in the past month, and a large percentage, 728 (75.5%), reported that their last purchase of antibiotics was from a community pharmacy.

**Table 1:** Sociodemographic characteristics of respondents

Sociodemographic	Frequency	Percent
Community Pharmacy (*N* = 964)		
Kubwa	213	22.1
Utako	119	12.3
Yaba	440	45.6
Lekki	103	10.7
Ajegunle	89	9.2
Sex (*N* = 962)		
Male	436	45.3
Female	526	54.7
Age (yrs.) (*N* = 961)		
18–24	189	19.7
25–34	358	37.3
35–44	242	25.2
45–54	106	11.0
55–64	41	4.3
Above 64	25	2.6
Education status (*N* = 955)		
No formal education	23	2.4
Primary	34	3.6
Secondary	201	21.0
Tertiary	697	73.0
Marital Status (*N* = 957)		
Single	462	48.3
Married	438	45.8
Divorced/Separated	32	3.3
Widowed	25	2.6
Occupation (*N* = 956)		
Unemployed	137	14.3
Self employed	314	32.8
Government Employed	165	17.3
Private Employed	309	32.3
Retired	31	3.2
Residence (*N* = 961)		
Rural	221	23.0
Urban	740	77.0
Religion (*N* = 955)		
Christianity	722	75.6
Islam	163	17.1
Others	70	7.3
Ethnic Affiliation (*N* = 953)		
Yoruba	421	44.2
Igbo	253	26.5
Hausa	82	8.6
Others	197	20.7
The last time antibiotic was used (*N* = 964)		
Last month	259	26.9
Last 3 months	206	21.4
Last 6 months	193	20.0
> 1 year	147	15.2
Can't remember	159	16.5
The last place antibiotic was retrieved (*N* = 959)		
Pharmacy	728	75.5
Patent medicine store/Chemist	107	11.1
Friend or family member	43	4.5
Can't remember	81	8.4
Antibiotic recommendation by healthcare professional (*N* = 936)		
Yes	640	68.4
No	187	20.0
Can't remember	109	11.6

The percentage of urban to rural community pharmacies used in this study was 60:40

Rural settings were areas with people of low socioeconomic status and poor access to basic amenities (Ajegunle and Kubwa)

When asked if they had sought counsel from a health professional before using an antibiotic, only 640 (68%) of the respondents agreed that they used antibiotics on the recommendation of a health professional. Most respondents correctly identified common antibiotics, such as metronidazole, ampicillin/cloxacillin, sulfamethoxazole/trimethoprim, and ciprofloxacin. However, 621 (64.4%) and 490 (50.8%) of the respondents wrongly identified chlorpheniramine and levonorgestrel as antibiotics, respectively (Table 2).

**Table 2:** Antibiotic identification by respondents (*N* = 964)

Antibiotic	Not Correct n (%)	Correct n (%)	Mean ± SD
Metronidazole	95 (9.9)	869 (90.1)	0.90 ± 0.29
Cotrimoxazole	84 (8.7)	880 (91.3)	0.91 ± 0.28
Levonorgestrel	490 (50.8)	474 (49.2)	0.49 ± 0.50
Ampicillin/Cloxacillin	37 (3.8)	927 (96.2)	0.96 ± 0.19
Tetracycline	33 (3.4)	931 (96.6)	0.97 ± 0.18
Ciprofloxacin	39 (4.0)	925 (96.0)	0.96 ± 0.19
Chlorpheniramine	621 (64.4)	343 (35.6)	0.36 ± 0.48

Among 964 responses on knowledge of antibiotic use and resistance (Table 3), 533 (55.3%) stated that bacteria caused malaria, and 491 (50.9%) reported that antibiotics could be used to relieve fever and pain. More than half of the respondents, 562 (58.3%), incorrectly specified that antibiotics are used immediately after unprotected sexual intercourse to prevent sexually transmitted diseases. 738 (76.6%) said bacteria would become less antibiotic-resistant after prolonged use.

**Table 3:** Respondents knowledge of antibiotic use and antibiotic resistance (*N* = 964)

Questions	Not correct *n* (%)	Correct *n* (%)	Mean (± SD)
Malaria is caused by bacteria	533 (55.3)	431 (44.7)	0.45 ± 0.47
Antibiotics are not prescribed for malaria	463 (48.0)	501 (52.0)	0.52 ± 0.50
Malaria and typhoid always occur together	471 (48.9)	493 (51.1)	0.51 ± 0.50
Antibiotics are prescribed for cough/cold as first choice of drug	456 (47.3)	508 (52.7)	0.53 ± 0.50
The body can fight mild infections on its own without antibiotics	262 (27.2)	702 (72.8)	0.73 ± 0.45
Antibiotics are not used immediately after unprotected sexual intercourse to prevent STDs	562 (58.3)	402 (41.7)	0.42 ± 0.49
Antibiotics cannot be used to prevent pregnancy	428 (44.4)	536 (55.6)	0.56 ± 0.49
Antibiotics can be used to relieve fever and pain	491 (50.9)	473 (49.1)	0.49 ± 0.50
Only antibiotics can stop stooling	422 (43.8)	542 (56.2)	0.56 ± 0.49
Hand hygiene can reduce the spread of bacterial infections	174 (18.0)	790 (82.0)	0.82 ± 0.39
Antibiotics resistance means that bacteria will not be killed by antibiotics	406 (42.1)	558 (57.9)	0.58 ± 0.49
If antibiotics are taken for a long period of time, bacteria can become less resistant to antibiotics	738 (76.6)	226 (23.4)	0.23 ± 0.42
Taking less than the prescribed dose can make bacteria more resistant	353 (36.6)	611 (63.4)	0.63 ± 0.48
Antibiotics have no side effect	352 (36.5)	612 (63.5)	0.63 ± 0.48
Physicians/pharmacists take time to provide information on the use of antibiotics	287 (29.8)	677 (70.2)	0.70 ± 0.46
I always use doctor’s prescription to purchase antibiotics from the pharmacy	359 (37.2)	605 (62.8)	0.63 ± 0.48

Table 4 highlights respondents’ attitudes toward antibiotic use. Over 293 (30%) of the respondents affirm that they expect antibiotics to be prescribed with an antimalarial. Strikingly, 213 (22.1%) construed that antibiotics cure their cold/sore throat faster, while 269 (27.9%) agreed that using leftover antibiotics was permissible when they had cold or similar symptoms. The analysis showed that 448 (46.5%) strongly agreed that mixing body creams with antibiotic cream was suitable for bleaching the skin of babies and adults.

**Table 4:** Respondents attitudes toward antibiotic use (*N* = 964)

Statements	Strongly disagree *n* (%)	Disagree *n* (%)	Neutral *n* (%)	Agree *n* (%)	Strongly agree *n* (%)	Mean ± SD
I expect antibiotics to be prescribed together with antimalaria	275 (28.5)	236 (24.5)	160 (16.6)	183 (19.0)	110 (11.4)	2.60 ± 1.370
The use of antibiotics prevents the symptoms of cold/sore throat from getting worse	62 (6.4)	115 (11.9)	145 (15.0)	358 (37.1)	284 (29.5)	3.71 ± 1.192
I believe antibiotics cure my cold/sore-throat faster	288 (29.9)	245 (25.4)	218 (22.6)	136 (14.1)	77 (8.0)	2.45 ± 1.268
It is permissible to take left-over antibiotics when I have cold or other related symptoms	127 (13.2)	151 (15.7)	185 (19.2)	269 (27.9)	232 (24.1)	3.34 ± 1.346
I can stop taking the prescribed antibiotics once I get better	171 (17.7)	175 (18.2)	127 (13.2)	210 (21.8)	281 (29.1)	3.26 ± 1.486
I can recommend prescribed antibiotics for family and friends for the same illness	137 (14.2)	195 (20.2)	141 (14.6)	208 (21.6)	283 (29.4)	3.32 ± 1.436
I prefer to run a laboratory test before taking antibiotics	21 (2.2)	76 (7.9)	215 (22.3)	256 (26.6)	396 (41.1)	3.96 ± 1.070
Antibiotics should not be used to boost or cleanse the immune system	106 (11.0)	133 (13.8)	211 (21.9)	215 (22.3)	299 (31.0)	3.49 ± 1.345
It is good to mix body creams with antibiotic creams to tone/bleach the skin for babies and adults	107 (11.1)	73 (7.6)	149 (15.5)	187 (19.4)	448 (46.5)	3.83 ± 1.376
All stooling should be stopped with an antibiotic	106 (11.0)	155 (16.1)	269 (27.9)	230 (23.9)	204 (21.2)	3.28 ± 1.268
I will get the antibiotics from another pharmacy if the pharmacist insists on not dispensing it	153 (15.9)	184 (19.1)	203 (21.1)	195 (20.2)	229 (23.8)	3.17 ± 1.397
Antibiotics should be used to prevent infections	284 (29.5)	264 (27.4)	201 (20.9)	113 (11.7)	102 (10.6)	2.47 ± 1.307
I prefer to take antibiotics with blood tonic, because they reduce blood volume	206 (21.40)	159 (16.5)	263 (27.3)	163 (16.9)	173 (17.9)	2.94 ± 1.380

**Fig. 1:**
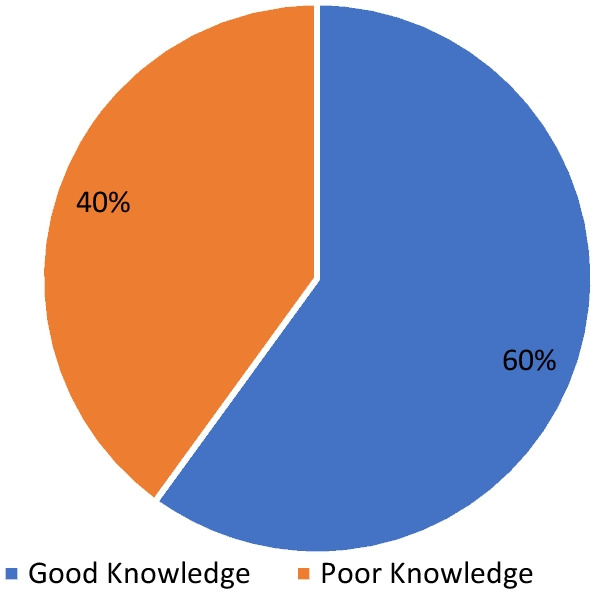
Respondents’ overall knowledge

**Fig. 2:**
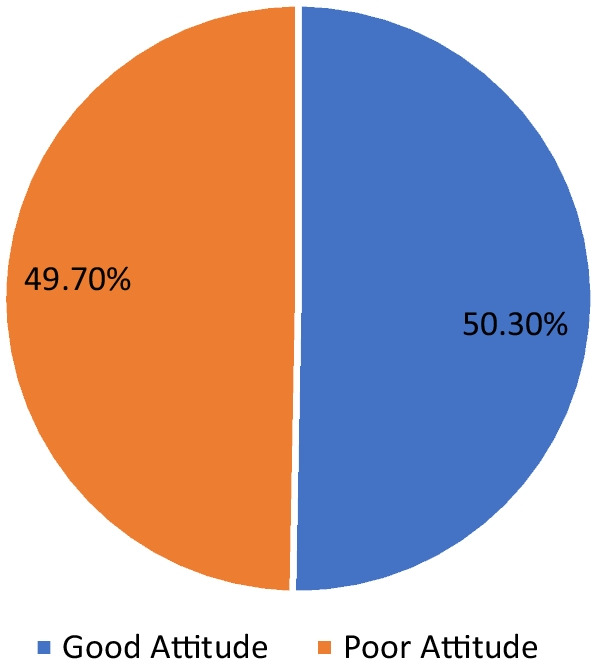
Respondents’ overall attitude

When grouped based on the mean score of 14.54 (± 3.41) for knowledge of antibiotics, 60.2% had good knowledge of antibiotic use. In comparison, 39.8% had poor knowledge (Fig. 1). For grouped attitude (Mean 41.81 ± 8.88), 50.3% had a good attitude toward antibiotics use, and 49.7% had poor attitude, an almost 50–50 relationship (Fig. 2).

Excluding sex and ethnic affiliation, a significant relationship exists between respondents’ knowledge and other sociodemographic predictors (*p* < 0.05) (Table 5) from the univariable logistic regression analysis.

**Table 5:** Predictors of good knowledge among the respondents with multivariable and univariable regression

Sociodemographic	Multivariable				Univariable	
	OR	95% CI		*p* value	OR	*p* value
		Lower	Upper			
Community pharmacy						
Kubwa (Reference)				.134		< .001*
Utako	1.62	.96	2.72	.067	1.37	0.169
Yaba	1.30	.86	1.95	.200	1.55	0.010*
Lekki	1.93	1.02	3.64	.041*	3.02	< .001*
Ajegunle	.91	.45	1.86	.813	0.46	0.003*
Sex						
Male (Reference)						
Female	1.16	.86	1.56	.329	1.16	0.241
Age (yrs.)						
18–24 (Reference)				.317		0.002*
25–34	1.54	1.01	2.36	.045*	1.88	< .001*
35–44	1.33	.80	2.20	.261	1.58	0.019*
45–54	1.96	1.02	3.76	.041*	2.38	0.001*
55–64	1.53	.59	3.94	.379	1.68	0.139
Above 64	1.29	.35	4.70	.697	3.4	0.012*
Education Status						
No formal education (Reference)				.044		< .001*
Primary	.42	.11	1.63	.214	0.70	0.534
Secondary	.30	.09	.97	.044*	1.01	0.978
Tertiary	.48	.15	1.50	.209	2.65	0.023*
Marital Status						
Single (Reference)				.114		0.035*
Married	1.32	.91	1.92	.137	1.33	0.037*
Divorced/Separated	.58	.24	1.37	.218	0.57	0.131
Widowed	1.89	.61	5.90	.268	1.56	0.307
Occupation						
Unemployed (Reference)				.101		< .001*
Self employed	.85	.53	1.36	.499	1.19	0.383
Government Employed	1.15	.66	2.02	.603	1.93	0.005*
Private Employed	1.43	.87	2.34	.154	2.91	< .001*
Retired	1.28	.38	4.25	.681	2.70	0.021*
Residence						
Rural						
Urban	1.45	1.01	2.08	.043*	1.92	< .001*
Religion						
Christianity (Reference)				.087		< .001*
Islam	.62	1.01	2.08	.027*	0.58	0.002*
Others	.85	1.01	2.08	.623	0.30	< .001*
Ethnic Affiliation						
Yoruba (Reference)				.344		0.079
Igbo	1.22	.83	1.78	.305	1.21	0.235
Hausa	1.32	.74	2.37	.344	0.70	0.147
Others	.87	.57	1.32	.520	0.80	0.220
Last time antibiotic was used (*N* = 964)						
Last month (Reference)				.068		0.031*
Last 3 months	1.16	.76	1.75	.477	1.34	0.118
Last 6 months	1.29	.84	1.99	.243	1.34	0.125
> 1 year	2.08	1.24	3.47	.005*	1.95	0.002*
Can't remember	1.51	.91	2.51	.111	1.09	0.666
Last place antibiotic was retrieved (*N* = 959)						
Pharmacy (Reference)				.243		< .001*
Patent medicine store/Chemist	.65	.40	1.06	.089	0.44	< .001*
Friend or family member	.59	.27	1.27	.180	0.29	< .001*
Can't remember	.92	.49	1.72	.815	0.58	0.022*
Health professional recommendation						
Yes (Reference)				.093		< .001*
No	.68	.46	1.01	.060	0.57	0.001*
Can't remember	.65	.39	1.09	.104	0.47	< .001*

* Significant at *p* < 0.05

**Fig. 3:**
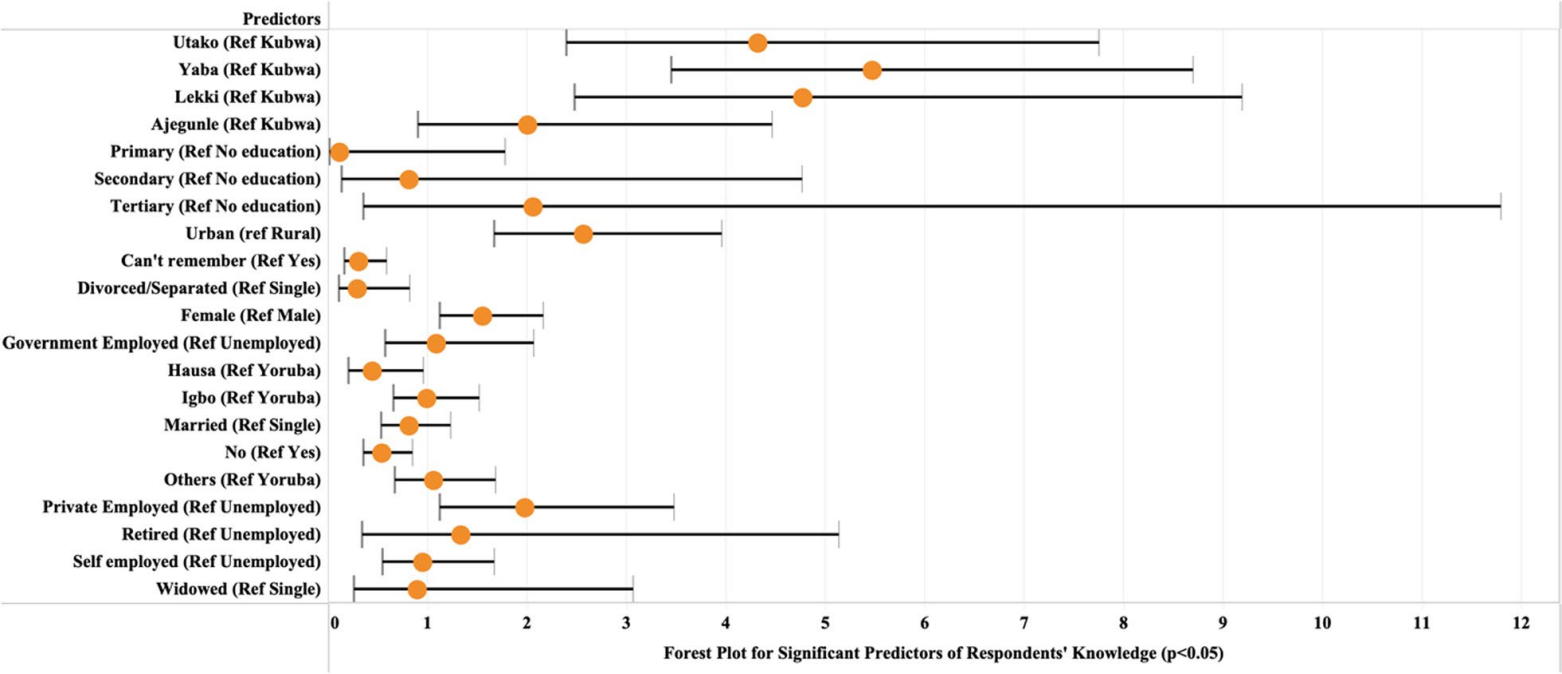
Predictors of respondents’ knowledge

Multivariable regression showed that factors, such as pharmacy location, residence, educational status, gender, and time the antibiotic was previously used had strong statistical associations with good knowledge. Respondents from the Lekki site in Lagos had over 93% greater odds (OR: 1.93, 95% CI 1.02–3.64) of having good knowledge about antibiotic use and resistance compared to respondents from Kubwa, Abuja. The odds of respondents between the age brackets of 25–34 (OR: 1.55, 95% CI 1.01–2.37) and 45–54 (OR: 1.97, 95% CI 1.03–3.76) having good knowledge of antibiotic were 1.547 and 1.966, respectively, relative to the reference age. In addition, urban residence had a strong statistical association with participants’ good knowledge of antibiotic use. Analysis showed that the odds of good knowledge on antibiotic use was 45% greater in urban residents (OR: 1.45, 95% CI 1.01–2.08) than in rural residents. Participants who took antibiotics for over 1 year (OR 2.08, 95% CI 1.25–3.47) had 2.08 greater odds of having good knowledge of antibiotic use and resistance than those who took it in less than a month (See Fig. 3).

Univariable analysis in Table 6 revealed that sociodemographic predictors included in this study were closely associated with respondents’ attitude (*p* < 0.05) except the period antibiotic was used and marital status.

**Table 6:** Predictors of good attitude among the respondents with multivariable and univariable regression

Sociodemographic	Multivariable				Univariable	
	OR	95% CI		*p* value	OR	*p* value
		Lower	Upper			
Community pharmacy						
Kubwa (Reference)				< .001*		< .001*
Utako	4.318	2.404	7.757	< .001*	2.915	< .001*
Yaba	5.478	3.449	8.700	< .001*	6.609	< .001*
Lekki	4.776	2.479	9.201	< .001*	11.327	< .001*
Ajegunle	2.006	.900	4.472	.089	1.340	0.314
Sex						
Male (Reference)						
Female	1.557	1.120	2.166	.008*	1.493	0.002*
Age (yrs.)						
18–24 (Reference)				.697		0.014*
25–34	1.349	.829	2.194	.228	1.490	0.028*
35–44	1.383	.781	2.452	.266	1.318	0.158
45–54	1.730	.845	3.541	.134	1.710	0.028*
55–64	1.809	.637	5.139	.266	1.298	0.451
Above 64	2.362	.519	10.759	.266	5.450	0.001*
Education status						
No formal education (Reference)				< .001		< .001*
Primary	.117	.008	1.776	.122	0.417	0.360
Secondary	.820	.141	4.774	.825	2.575	0.139
Tertiary	2.067	.362	11.798	.414	10.169	< .001*
Marital Status						
Single (Reference)				.136		0.060
Married	.818	.540	1.239	.343	0.862	0.265
Divorced/Separated	.293	.104	.826	.020*	0.347	0.009*
Widowed	.902	.265	3.070	.869	0.960	0.920
Occupation						
Unemployed (Reference)				.006		< .001*
Self employed	.959	.549	1.677	.884	1.300	0.219
Government Employed	1.094	.578	2.067	.783	1.557	0.063
Private Employed	1.979	1.126	3.478	.018*	4.447	< .001*
Retired	1.331	.344	5.145	.678	3.032	0.007*
Residence						
Rural						
Urban	2.574	1.673	3.961	< .001*	4.290	< .001*
Religion						
Christianity (Reference)				.329		< .001
Islam	.768	.475	1.242	.282	0.523	< .001*
Others	.610	.262	1.423	.253	0.260	< .001*
Ethnic Affiliation (*N* = 953)						
Yoruba (Reference)				.189		< .001*
Igbo	.999	.657	1.517	.995	0.792	0.145
Hausa	.450	.211	.958	.038*	0.185	< .001*
Others	1.067	.675	1.687	.782	0.739	0.080
Last time antibiotic was used (*N* = 964)						
Last month (Reference)				.807		0.092
Last 3 months	.936	.590	1.484	.778	1.136	0.495
Last 6 months	.929	.573	1.507	.765	1.170	0.409
> 1 year	1.192	.690	2.058	.529	1.777	0.006*
Can't remember	1.245	.695	2.230	.462	1.088	0.677
Last place antibiotic was retrieved (*N* = 959)						
Pharmacy (Reference)				.428		< .001*
Patent medicine store/Chemist	.695	.389	1.240	.218	0.263	< .001*
Friend or family member	.603	.236	1.542	.291	0.287	< .001*
Can't remember	.734	.351	1.533	.410	0.331	< .001*
Health professional recommendation						
Yes (Reference)				< .001		< .001*
No	.547	.351	.853	.008*	0.360	< .001*
Can't remember	.311	.165	.584	< .001*	0.182	< .001*

*Significant at *p* < 0.05

**Fig. 4:**
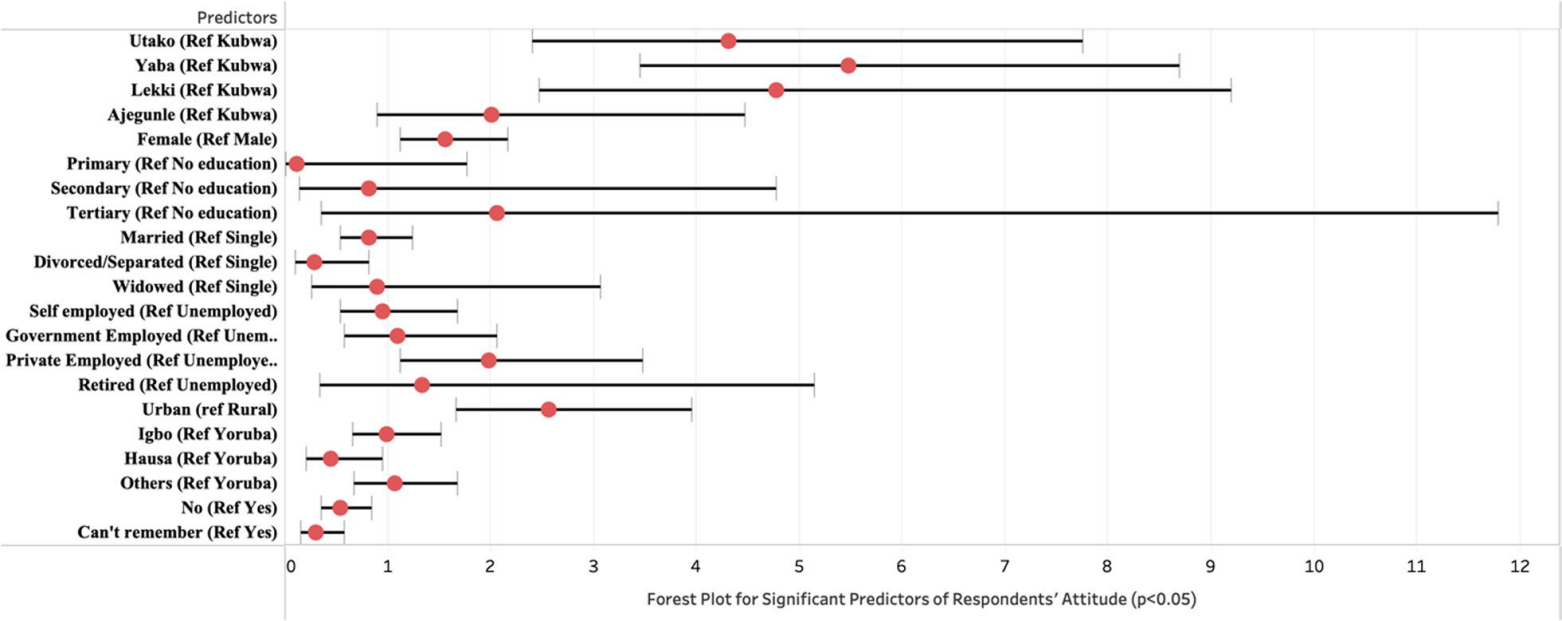
Predictors of respondents’ attitudes

Some predictors showed a significant relationship with participants' attitudes toward antibiotic use with the multivariable logistic regression (Table 6.). Among the respondents from the five community pharmacies, participants from Yaba (OR: 5.48, 95% CI 3.45–8.70) and Lekki (OR 4.78, 95% CI 2.48–9.20) community pharmacies in Lagos have 5.48 and 4.78 greater odds of having good attitude on antibiotic use compared to participants from Kubwa in Abuja. Females (OR: 1.56, 95% CI 1.12–2.16) showed 55% greater likelihood of having good attitudes toward antibiotic use and resistance reference to males. Participants living in urban areas have 157% greater odds (OR: 2.57, 95% CI 1.67–3.96) of having a good attitude toward antibiotic use and resistance compared to rural participants. The analysis also reveals that participants who took their last antibiotic without a recommendation from a health professional (OR: 0.55, 95% CI 0.35–0.85) have 46% likelihood of having good attitude toward antibiotic use in reference to those that got a recommendation from a health professional (Fig. 4).

## Discussion

Few studies in Nigeria have concurrently evaluated community pharmacy clients' knowledge and attitude toward antibiotic use and resistance in different regions. Previous studies on antimicrobial resistance were restricted to single locations, such as tertiary hospitals, rural communities, or community pharmacies in a state [17–20]. This assessment considers randomly selected community pharmacies in Abuja and Lagos, the two major metropolises in Nigeria's multiculturally diverse population. In addition, the modified questions used in this survey were deductively generated and informed by community pharmacists' interactions with patients.

In a multi-country antimicrobial survey, the WHO estimated that over one-third of the participants took antibiotics within the past month. Egypt topped the list, while nearly half of Nigeria’s participants attested to taking antibiotics within the last month [14]. Similarly, most respondents in this study reported taking antibiotics in the last month, but respondents who last took an antibiotic over a year tend to have more knowledge about antibiotics. In addition, nearly, half of the participants in a study to assess public awareness of antimicrobial resistance in Nigeria also attest to having taken antibiotics in the last month [21]. There is a need to increase awareness of antibiotic abuse and misuse through effective public campaigns. Strict laws on antibiotic prescriptions must be enacted to implement a prescription-only policy and efficiently monitor the methods associated with dispensing antibiotics in community pharmacies. Nigeria's medication distribution system is also responsible for medication abuse due to the open-drug system allowing uncontrolled and undefined access to medications from patent medicine stores whose longer open hours, low cost, and shorter waiting times make them more accessible [22]. Hence, patent medicine dealers must be incorporated into the campaign to improve public misuse of antibiotics if the government cannot abolish the poorly regulated medication market.

Most of our respondents purchased antibiotics from pharmacies, which allows pharmacists to interact with and educate patients on antibiotic use. Such intervention can be explored when enacting policies toward curbing increasing resistant microbes. This strategy has been proven to improve public knowledge and perception of antibiotic use and resistance [23].

The results on antibiotics identification are consistent with similar findings in a study among civil servants in Uyo, Nigeria, where the five most commonly used antibiotics reported were ampicillin/cloxacillin, metronidazole, sulfamethoxazole/trimethoprim, ciprofloxacin and amoxicillin [24]. Empirical evidence from a local population in Nigeria showed that ampicillin/cloxacillin was the most common antibiotic used for self-medication. Others were amoxicillin, ampicillin, tetracycline, metronidazole, and ciprofloxacin [19]. These selected antibiotics will have high levels of resistant microbes due to easy access and unscrupulous use by the public [18, 25]. In general, respondents showed good knowledge of antibiotics and antibiotic resistance. However, over half of the participants believed that bacteria cause malaria and that antibiotics are prescribed for malaria—a gross misconception from the public and could explain the pressure physicians face to prescribe antibiotics [26].

A previous study showed that some people take antibiotics before and after sex to prevent sexually transmitted infections [27]. Strikingly, most of our respondents said that antibiotics are used to prevent sexually transmitted diseases after unprotected sexual intercourse. It also explains why many study participants chose levonorgestrel as an antibiotic, because it is majorly used after sex. This unscrupulous use of antibiotics has abetted the growing number of resistant bacteria in clinical settings. In the present study, half of the respondents would take antibiotics to relieve fever and pain, which was similar to a study whose participants thought antibiotics could relieve most cold symptoms [28]. Some participants believe antibiotics are less resistant with increasing duration of use. On the contrary, a similar study showed that respondents’ claimed that bacteria become more resistant to an increase in the duration of antibiotic use [13].

There is little difference between the participant’s good and poor attitudes toward antibiotic use. The belief that antibiotics could cure symptoms of cold and sore throat faster and their ability to prevent symptoms of cold from getting worse correlates with a study, where majority showed a negative attitude using antibiotics for cough, cold and skin wounds [29]. A similar response to antibiotics attitude was recorded in a previous survey, where majority believed taking antibiotics when having a cold made them recuperate faster [30]. Frequent prescribing of antibiotics for viral respiratory infections, which could be self-limiting, has influenced public thought on the effectiveness of antibiotics in treating these illnesses. The right approach to change this belief would be to increase sensitization on common viral infections and their differences from bacterial infections.

Antibiotic creams are widely misused for bleaching/toning skin color in Nigeria. Popularly branded triple-action creams by pharmaceutical companies, antimicrobial creams contain antifungal, antibacterial, and steroidal agents and are readily available over the counter in Nigeria. It is a popular culture among community pharmacy clients in Nigeria to mix and use triple actions creams with regular body creams to enhance their skin tone. Hence, most participants strongly agreed that it is good to mix body creams with antibiotic creams to tone/bleach the skin for babies and adults, as seen in this study. The ease of obtaining antibiotics over the counter in developing countries is also a significant concern [12]. Almost 80% of all pharmaceuticals in developing countries are dispensed by individuals without formal training and certification [31]. A high proportion of the respondents strongly posited that they would retrieve the antibiotic from another supplier if the pharmacist insisted on not dispensing it.

The findings from the univariate and multivariable analyses supported the study’s hypothesis that community pharmacy patients’ characteristics are predictors of knowledge and attitude toward antibiotic use. Educational status was among the predictors of respondents’ knowledge on antibiotic use and resistance. This is consistent with a study conducted in Jos, Nigeria, among consumers of antibiotics in community pharmacies [17]. With a higher level of education among clients of community pharmacies, it will be easy to control the abuse of antibiotics and effectively educate them on the dangers of antimicrobial resistance. On the contrary, higher levels of education have been shown to influence self-medication with antibiotics for the treatment of ailments which can lead to the emergence of resistant organisms [19]. Some patients may be self-assured that they have sufficient knowledge of medication use, because they can read and understand the label information and probably get more information on the internet.

Lekki is a suburb of Lagos with people of high economic status and levels of education. It was no surprise to see higher odds of good attitude and knowledge toward antibiotic use, because the area possesses top-notch health facilities with qualified health professionals in Lagos. In addition, this location is also an urban residence which resonates with the result of the analysis which showed that respondents living in urban areas are more likely to have good attitudes and knowledge about antimicrobial resistance and use.

Attitudes toward antibiotic use between male and female participants have shown conflicting evidence in previous studies [32]. This study established that females are more likely to have a good attitude toward antibiotic use than their male counterparts. Poor attitude toward antibiotics can lead to misuse in either gender. A study has shown that women in Nigeria discriminately use antibiotics for symptoms that do not require empiric bacteria treatment, such as menstrual symptoms [33].

## Conclusion

The study evaluated the knowledge and attitude of respondents to antibiotic use in two major cities in Nigeria and, at the same time, compared it to their sociodemographic factors. A high percentage of participants showed good knowledge of antibiotic use, while the percentage difference between poor and good attitude was significantly small. Greater efforts need to be channeled into antimicrobial resistance campaign to address the misuse and abuse of antibiotics. It revealed that health professionals, especially community pharmacists have active roles to play, because they are at the epicenter of the medication distribution system and should uphold strict antibiotic stewardship when attending to patients. Analysis established that factors such as educational status, age, gender, and residence have strong statistical association on respondents’ knowledge and attitude to antibiotic use. To this end, educational institutions are one of the best settings to carry out antibiotic sensitization programs to educate young people on the appropriate and rational use of antibiotics and the dangers associated with indiscriminate use. Health policymakers could leverage on this information to enact laws and create programs that will reduce the abuse and misuse of antibiotics in the hospital and community pharmacy settings.

## Limitations

This observational study is cross-sectional and comes with its limitations. Close associations between predictors and respondents’ attitude and knowledge do not mean causality, because data were collected once for each participant. Though the study population was drawn from five community pharmacies in different geographical locations in Nigeria, the findings do not generalize to other community pharmacy clients. There might be variations in findings if a similar study was done in other parts of the country. Respondents were prone to recall bias when asked about the last time and last place antibiotic was used and retrieved, respectively. It was reduced by increasing the interval between each sub-variables under the time and place predictors and aggregating them as categorical variables.

## Data Availability

Data are available on https://doi.org/10.6084/m9.figshare.21150418.v1

## Abbreviations

WHO: World Health Organization
OR: Odds ratio
CI: Confidence interval
